# Clinicopathological Spectrum of Pediatric Renal Biopsies: A Retrospective Observational Study From South India

**DOI:** 10.7759/cureus.114894

**Published:** 2026-08-20

**Authors:** Anil Kumar H, Vinay K S, Somanath N

**Affiliations:** 1 Pediatrics, Bangalore Medical College and Research Institute, Bengaluru, IND; 2 Pathology, Institute of Nephrourology, Bengaluru, IND

**Keywords:** complement c3/c4, histopathological patterns, infection-related glomerulonephritis, lupus nephritis, nephrotic syndrome, pediatric renal biopsy

## Abstract

Background: Kidney disorders in Indian children remain incompletely characterized, with impaired renal function affecting a notable proportion of the pediatric population. Renal biopsy remains underutilized, as childhood nephrotic syndrome is often presumed to reflect minimal change disease (MCD), and no national pediatric renal biopsy registry exists. This study addresses this gap by examining the indications for renal biopsy and the histopathological patterns observed in a South Indian cohort. The study aimed to determine the epidemiological profile, clinical indications, and histopathological spectrum of pediatric renal biopsies and to assess their correlation with selected clinical and laboratory parameters.

Materials and methods: This retrospective observational study, conducted from January 2023 to February 2026, included children aged ≤18 years who underwent native renal biopsy at a tertiary care center. Clinical and laboratory data, including hemoglobin, serum creatinine, serum C3 and C4 levels, urine protein-to-creatinine ratio, and urinalysis findings, were retrieved from case records and the laboratory information system. Ultrasound-guided renal biopsies were performed using 16- or 18-gauge needles, with two cores obtained for light microscopy and immunofluorescence. Immunofluorescence staining was performed for IgG, IgA, IgM, C3, C1q, kappa, and lambda, and the results were evaluated by a nephropathologist. Inadequate biopsy specimens containing fewer than seven glomeruli were excluded.

Results: Among the 54 pediatric renal biopsies, 66.7% were from female patients (M/F ratio, 1:2), and the mean age was 10.77 ± 4.54 years. Nephrotic syndrome was the leading indication for biopsy (50%, n = 27), followed by nephritic syndrome (24.07%, n = 13). Secondary glomerulonephritis (GN) predominated (57.41%), with lupus nephritis (LN) being the most frequent diagnosis (37%, n = 20), followed by infection-related GN (IRGN) (16.7%, n = 9) and MCD/focal segmental glomerulosclerosis (13% each, n = 7). Among patients with LN, Class IV was the predominant class (50%, n = 10), followed by Class II (30%, n = 6). Serum C3 and C4 levels differed significantly across diagnostic categories (both p < 0.001), with consistently low levels observed in patients with LN and IRGN.

Conclusions: Nephrotic syndrome remains the leading indication for pediatric renal biopsy, with secondary GN, particularly LN and IRGN, predominating in this cohort, in contrast to patterns reported in Western studies. LN was the most frequent histopathological diagnosis, with Class IV LN accounting for half of the LN cases. Complement levels varied significantly across diagnostic categories, with low C3 and C4 levels observed in LN, consistent with immune complex-mediated complement consumption. These findings reinforce the importance of renal biopsy in establishing accurate diagnoses and guiding the management of pediatric renal diseases.

## Introduction

The burden of chronic kidney disease in children and adolescents is not well characterized in India. However, according to the Comprehensive National Nutrition Survey, impaired kidney function affects approximately 4% of Indian children and adolescents [1-2]. Renal biopsy is relatively uncommon in children compared with adults [3]. One reason for this is that childhood nephrotic syndrome is most commonly presumed to be due to minimal change disease (MCD), and corticosteroid therapy is initiated empirically [4].

Nephrotic syndrome is the most common glomerular disorder in the pediatric population, with an incidence ranging from 1.15 to 16.9 per 100,000 children. Renal biopsy is not necessary in most cases for diagnosis or treatment. However, biopsy may be indicated at presentation in children with atypical features, such as gross hematuria, decreased serum C3 levels, acute kidney injury (AKI) unrelated to hypovolemia, sustained hypertension, arthritis, and/or rash. In certain disorders, such as lupus nephritis (LN) and IgA nephropathy (IgAN), the severity of renal involvement cannot be determined solely from clinical findings, necessitating a renal biopsy for accurate assessment [5-6].

In children with inherited nephropathies and chronic hematuria, renal biopsy can serve as an important tool for establishing the diagnosis and determining prognosis [5]. Renal biopsy is a well-established and invaluable diagnostic procedure for evaluating kidney diseases. It provides diagnostic information, assists in selecting appropriate therapeutic strategies for many renal disorders, and offers prognostic information. The spectrum of renal diseases in children differs from that in adults. Although renal diseases are relatively uncommon in the pediatric population, they can result in substantial morbidity, making accurate diagnosis essential for appropriate treatment and prognostication [7-8].

The most common indications for pediatric renal biopsy include nephrotic syndrome, particularly steroid-resistant and atypical forms, nephritic syndrome, persistent hematuria, and AKI [1]. Although renal biopsies are performed in children and reported by various centers across India, no national renal biopsy registry currently exists to characterize the epidemiology and prevalence of kidney diseases and their regional variations. This gap has been partially addressed by data from single-center and multicenter renal biopsy studies. This study provides insights into the indications for renal biopsy, histopathological patterns, and the spectrum of kidney diseases in the pediatric population while contributing data from South India.

We aimed to determine the epidemiological profile, clinical indications, and histopathological spectrum of pediatric renal biopsies and to assess their correlation with selected clinical and laboratory parameters.

## Materials and methods

Study design and setting

This retrospective observational study was conducted from January 2023 to February 2026 at Vani Vilas Hospital, Bangalore Medical College and Research Institute, and the Institute of Nephrourology, Bengaluru. Clinical and laboratory data were obtained from case files and the laboratory information system (LIS), respectively. Ethical approval was obtained from the Institutional Ethics Committee of Bangalore Medical College and Research Institute (approval number: BMCRI/EC/11/2026).

Inclusion and exclusion criteria

Children aged ≤18 years who were admitted with kidney disease and underwent native renal biopsy were included in the study. Renal biopsy specimens containing fewer than seven glomeruli, only medullary tissue, or only fat, connective tissue, or skeletal muscle were excluded. Specimens without a core available for immunofluorescence microscopy were also excluded. Post-transplant renal biopsies and repeat biopsies from the same patient were excluded.

Clinical and laboratory data collection

Clinical data, including age, sex, clinical presentation, symptom duration, and treatment history, were collected from case files. Laboratory parameters were retrieved from the LIS. They included hemoglobin (sodium lauryl sulfate method), serum creatinine (enzymatic method), serum C3 and C4 levels (immunoturbidimetric method), urine protein-to-creatinine ratio (UPCR), and urinalysis findings. UPCR was calculated using urine protein measured by the benzethonium method and urine creatinine measured by the enzymatic method. Urinalysis findings, including proteinuria and hematuria, were obtained using the UriSed 3 Pro and LabUMat 2 automated microscopy and chemistry analyzers (77 Elektronika Kft., Budapest, Hungary).

Renal biopsy

All renal biopsies were performed by a pediatric nephrologist under real-time ultrasound guidance with mild sedation and local anesthesia using a 16- or 18-gauge biopsy gun after obtaining informed consent from the parents or guardians. Two passes were performed to obtain two renal cores. One core was placed in 10% formalin for light microscopy, while the other was placed in normal saline or Michel's transport medium for immunofluorescence microscopy.

Histopathological processing

The formalin-fixed renal core was routinely processed and paraffin-embedded. Multiple 3-µm sections were prepared and stained with hematoxylin and eosin (H&E), periodic acid-Schiff (PAS), Jones methenamine silver, and Masson trichrome stains. The stained sections were examined by light microscopy.

Immunofluorescence studies

Renal cores submitted for immunofluorescence in normal saline were cryo-embedded and sectioned at 3 µm thickness using a cryostat. The frozen sections were washed with phosphate-buffered saline and treated with fluorescein-conjugated antibodies against IgG, IgA, IgM, C3, C1q, kappa, and lambda. The sections were then examined under a fluorescence microscope using a blue filter.

Histopathological evaluation

All renal biopsy specimens were evaluated and reported by an experienced nephropathologist based on light microscopy and immunofluorescence findings. On light microscopy, the following parameters were recorded: total number of glomeruli; glomerular proliferation and pattern of injury; segmental and global glomerulosclerosis; degree of interstitial fibrosis and tubular atrophy; and vascular changes. On immunofluorescence, the location of deposits (capillary, mesangial, or both), staining pattern (granular or linear), and staining intensity (0-4) were recorded. Renal biopsy interpretation was based on light microscopy, immunofluorescence, and clinicopathological correlation using standard diagnostic criteria.

Diagnostic criteria for glomerulonephritis (GN)

Lupus Nephritis (LN)

LN was diagnosed based on light microscopic features, full-house immunofluorescence staining, and positive autoimmune serology. Cases were classified according to the International Society of Nephrology/Renal Pathology Society (ISN/RPS) classification, and disease activity and chronicity were assessed using the modified National Institutes of Health (NIH) activity and chronicity indices.

IgA Nephropathy (IgAN)

IgAN was diagnosed based on light microscopic findings and predominant or codominant mesangial IgA deposition on immunofluorescence microscopy. Cases were classified according to the Oxford MEST-C classification.

Focal Segmental Glomerulosclerosis (FSGS)

FSGS was diagnosed only when segmental sclerosis was identified on light microscopy. Cases were classified into histological variants according to the Columbia classification.

Minimal Change Disease (MCD)

MCD was defined by normal glomerular morphology on light microscopy, negative immunofluorescence for immune deposits, and compatible clinical features of nephrotic syndrome or proteinuria. In the absence of electron microscopy, the diagnosis was based on clinicopathological findings.

Immune Complex Glomerulonephritis (ICGN)

Cases demonstrating granular glomerular deposits of immunoglobulins and complement on immunofluorescence, without serological evidence of systemic lupus erythematosus (SLE) (negative antinuclear antibody (ANA) and anti-double-stranded DNA antibodies (anti-dsDNA)), postinfectious GN (negative antistreptolysin O titer, where clinically indicated), or other identifiable secondary causes, were classified as ICGN.

Cases showing C3-dominant immunofluorescence without electron microscopic evaluation were classified as infection-related GN (IRGN) based on clinicopathological correlation. A definitive diagnosis of C3 glomerulopathy was not made in the absence of ultrastructural confirmation.

Patient classification

Patients were assigned to a single, mutually exclusive cohort based on a predefined clinical hierarchy of their primary presentation to ensure nonoverlapping categorization.

Definitions

Hematuria

Hematuria was defined as either gross hematuria, characterized by reddish urine, or microscopic hematuria, defined as >5 red blood cells (RBCs) per high-power field in a centrifuged urine sample [5].

Nephrotic-Range Proteinuria

Nephrotic-range proteinuria was defined as a UPCR of ≥2 g/g in a spot urine sample or proteinuria of ≥1,000 mg/m² per day in a 24-hour urine sample [6].

Nephrotic Syndrome

Nephrotic syndrome was defined as massive proteinuria (UPCR ≥2 g/g in a spot urine sample or ≥1,000 mg/m² per day in a 24-hour urine sample) accompanied by hypoalbuminemia (serum albumin <3 g/dL) or generalized edema [6].

Steroid-Resistant Nephrotic Syndrome (SRNS)

SRNS was defined as failure to achieve complete remission within four weeks of treatment with daily prednisone or prednisolone at the standard dose [6].

Acute Kidney Injury (AKI)

The AKI indication cohort was strictly reserved for patients presenting with unexplained, rapidly deteriorating renal function without a dominant classical nephrotic or nephritic presentation, such as isolated acute tubular injury or suspected pigment cast nephropathy (PCN) in the presence of non-nephrotic proteinuria and inactive urinary sediment. Patients with LN or IRGN who presented with AKI were categorized under the primary glomerular syndromes of nephritic or nephrotic/nephritic syndrome because they fulfilled the diagnostic criteria for these syndromes, including active urinary sediment, hematuria, hypertension, and severe proteinuria.

Hypertension

Blood pressure (BP) in children was classified according to age-based pediatric BP reference charts. Hypertension was defined as an average systolic or diastolic BP ≥ the 95th percentile for age, sex, and height [9].

Nephritic Syndrome

Nephritic syndrome was defined by the presence of gross or microscopic hematuria, hypertension, oliguria, edema, and reduced glomerular filtration rate [7].

Statistical methods

Data were analyzed using SPSS Statistics version 27.0 (IBM Corp. Released 2020. IBM SPSS Statistics for Windows, Version 27.0. Armonk, NY). Data distribution and normality were assessed by visual inspection of Q-Q plots and numerical assessment of skewness. Variables with skewness values between −2 and +2 were considered approximately normally distributed. Continuous variables are presented as mean ± standard deviation (SD), whereas categorical variables are presented as frequencies and percentages. Fisher's exact test was used to assess associations between categorical variables. The Kruskal-Wallis test was used to compare groups. Post hoc pairwise comparisons were not performed because the sample size was insufficient to support multiple-comparison adjustments. A p-value <0.05 was considered statistically significant.

## Results

Of the 975 renal biopsies performed during the study period, 54 (5.54%) were obtained from pediatric patients. Of these, 18 (33.3%) were from male patients and 36 (66.7%) were from female patients, yielding a male-to-female ratio of 1:2. The mean age of the patients was 10.77 ± 4.54 years. The clinical and laboratory profiles of the study cohort are presented in Table 1.

**Table 1:** Summary of clinical and laboratory findings in the study cohort Continuous variables are presented as mean ± SD. Categorical variables are presented as frequencies (n) and percentages (%). UPCR: urine protein-to-creatinine ratio, SD: standard deviation, n: number, %: percentage

Variable	Value
Age in years (mean ± SD)	10.77 ± 4.54
Sex, n (%)	Male: 18 (33.3%)
	Female: 36 (66.7%)
Serum creatinine mg/dL (mean ± SD)	1.47 ± 1.1
UPCR (mean ± SD)	5.54 ± 4.25
Serum C3 mg/dL (mean ± SD)	56.19 ± 47.5
Serum C4 mg/dL (mean ± SD)	20.79 ± 13.9
Hypertension (n = 46)	Absent: 5 (11%)
	Present: 41 (89%)
Urine protein	Nil: 3 (5.6%)
	1+: 7 (13%)
	2+: 12 (22.2%)
	3+: 20 (37%)
	4+: 12 (22.2%)
Hematuria (n = 49)	Present: 38 (70.4%)
	Absent: 11 (20.4%)

**Figure 1:**
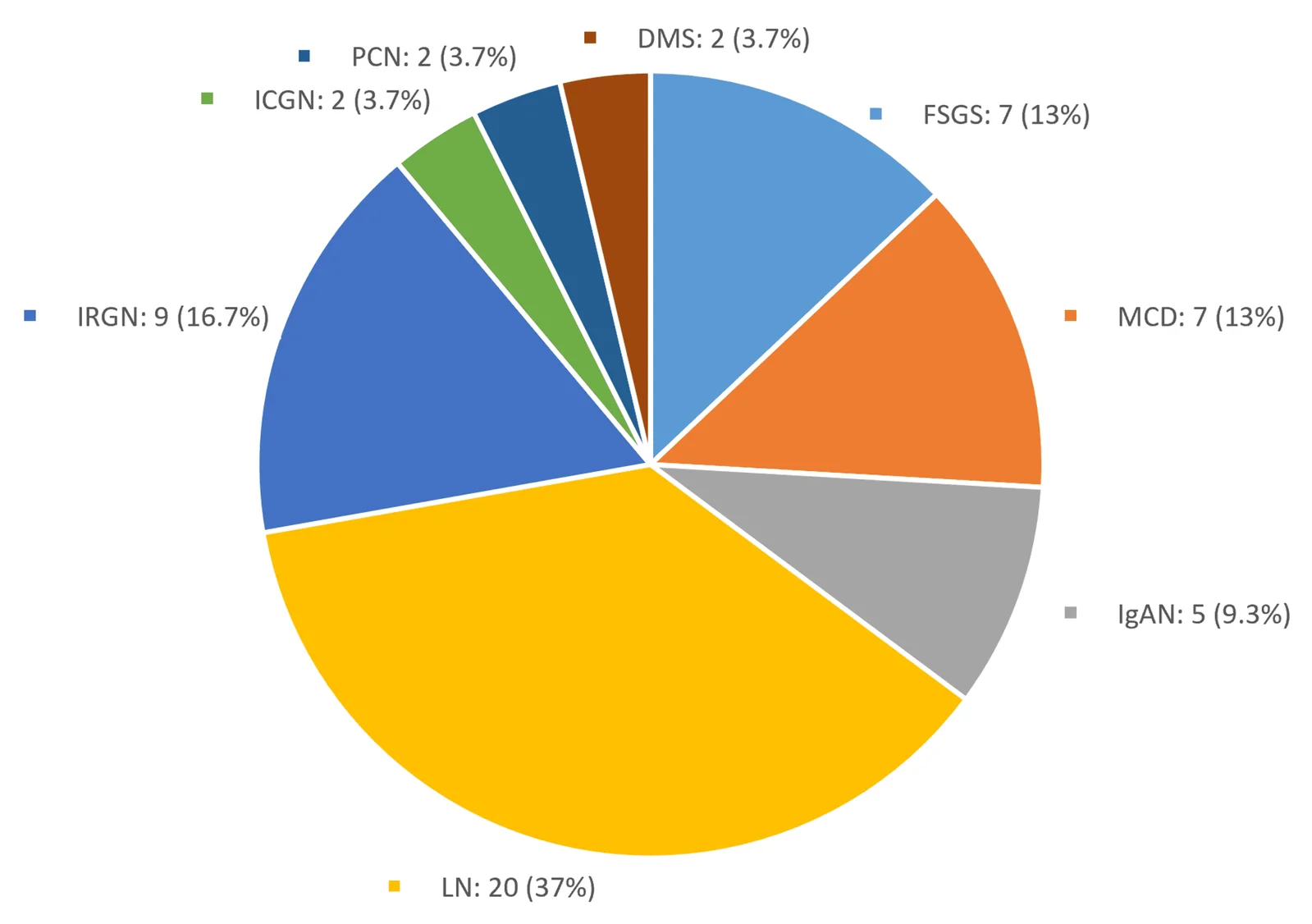
Prevalence of various histological lesions observed in the study cohort (n = 54) Data are presented as frequencies (n) and percentages (%). FSGS: focal segmental glomerulosclerosis, MCD: minimal change disease, DMS: diffuse mesangial sclerosis, IRGN: infection-related glomerulonephritis, ICGN: immune complex glomerulonephritis, LN: lupus nephritis, IgAN: IgA nephropathy, PCN: pigment cast nephropathy

Primary GN was observed in 21 cases (38.88%), secondary GN in 31 cases (57.41%), and tubulointerstitial disease in 2 cases (3.70%). MCD (n = 7, 13%) and FSGS (n = 7, 13%) were observed exclusively in patients with atypical clinical presentations. Among the seven patients with MCD, the indications for renal biopsy were SRNS (n = 3), nephrotic syndrome with hypertension as an atypical feature (n = 3), and nephrotic syndrome with gross hematuria as an atypical feature (n = 1). IRGN was the most common diagnosis among primary GN cases, whereas LN was the most common diagnosis among secondary GN cases. Of the nine patients with IRGN, antistreptolysin O titer results were available for seven (77.8%); five (55.55%) were positive, two (22.22%) were negative, and testing was not performed in two (22.22%). Among patients with LN, ANA and anti-dsDNA antibodies were positive in 18 cases (90%) and borderline positive in two cases (10%). The frequencies of the different histopathological diagnoses are presented in Figure 1.

The most common indication for renal biopsy was nephrotic syndrome, accounting for 27 cases (50%), followed by nephritic syndrome, with 13 cases (24.07%). The histopathological diagnoses and their corresponding clinical presentations in the study cohort are presented in Table 2.

**Table 2:** Clinical presentations/indications for biopsy and histopathological diagnoses in the study cohort (n = 54) Data are expressed as frequencies (n) and percentages (%) FSGS-NOS: focal segmental glomerulosclerosis, not otherwise specified, MCD: minimal change disease, DMS: diffuse mesangial sclerosis, IRGN: infection-related glomerulonephritis, AKI: acute kidney injury, LN: lupus nephritis, IgAN: IgA nephropathy, ICGN: immune complex glomerulonephritis, PCN: pigment cast nephropathy

Histological diagnosis	Nephrotic syndrome n (%)	Nephritic syndrome n (%)	Nephrotic/nephritic syndrome n (%)	AKI n (%)
					FSGS-NOS	6 (11.11%)	1 (1.85%)	0 (0%)	0 (0%)
MCD	7 (12.96%)	0 (0%)	0 (0%)	0 (0%)
IgAN	3 (5.55%)	0 (0%)	2 (3.70%)	0 (0%)
LN	6 (11.11%)	7 (12.96%)	7 (12.96%)	0 (0%)
IRGN	2 (3.70%)	5 (9.25%)	1 (1.85%)	1 (1.85%)
ICGN	1 (1.85%)	0 (0%)	1 (1.85%)	0 (0%)
PCN	0 (0%)	0 (0%)	0 (0%)	2 (3.70%)
DMS	2 (3.70%)	0 (0%)	0 (0%)	0 (0%)
Column total	27 (50%)	13 (24.07%)	11 (20.37%)	3 (5.56%)

The laboratory parameters, according to the histopathological diagnosis, are presented in Table 3. Fisher’s exact test was used to assess associations between categorical variables, while the Kruskal-Wallis test was used for comparisons across groups. A p-value <0.05 was considered statistically significant.

**Table 3:** Comparison of laboratory parameters with the histological diagnosis *Statistically significant p-value. Continuous variables are presented as mean ± SD. Categorical variables are presented as frequencies (n) and percentages (%). Fisher’s exact test was used to analyze the association between categorical variables. The Kruskal-Wallis test was used for group comparisons. Results were considered to be statistically significant if the p-value was < 0.05. DMS: diffuse mesangial sclerosis, FSGS: focal segmental glomerulosclerosis, IgAN: IgA nephropathy, IRGN: infection-related glomerulonephritis, LN: lupus nephritis, MCD: minimal change disease, SD: standard deviation, ICGN: immune complex glomerulonephritis, PCN: pigment cast nephropathy

Variable			DMS	FSGS	IgAN	ICGN	IRGN	LN	MCD	PCN	p-value
No. of cases n (%)			2 (3.7%)	7 (13%)	5 (9.3%)	2 (3.7%)	9 (16.7%)	20 (37%)	7 (13%)	2 (3.7%)	NA
												Age in years (mean ± SD)			1 ± 1.4	7.57 ± 5.38	8.6 ± 3.2	12.5 ± 4.95	10.77 ± 3.15	14.25 ± 1.99	8.28 ± 3.94	9.5 ± 4.9	<0.001*
Sex n (%)		M	0 (0%)	5 (71.4%)	2 (60%)	1 (50%)	5 (55.6%)	3 (15%)	2 (28.6%)	0 (0%)	0.07
		F	2 (100%)	2 (28.6%)	3 (40%)	1 (50%)	4 (44.4%)	17 (85%)	5 (71.4%)	2 (100%)	
Serum creatinine mg/dL (mean ± SD)			1.95 ± 0.07	0.72 ± 0.73	1.01 ± 0.5	2.11 ± 0.73	1.36 ± 0.6	1.91 ± 1.31	0.90 ± 1.28	2.05 ± 0.21	0.017*
Serum C3 mg/dL (mean ± SD)			99.5 ± 2.12	95.63 ± 4.86	35.9 ± 27.19	33.4 ± 5.09	38.15 ± 40.98	37.45 ± 20.63	94.57 ± 8.64	82.7 ± 2.51	<0.001*
Serum C4 mg/dL (mean ± SD)			40.75 ± 0.77	31.08 ± 7.09	19.67 ± 11.48	5.49 ± 1.82	21.07 ± 13.64	11.06 ± 10.93	32.78 ± 5.98	37 ± 1.41	<0.001*
UPCR			11.6 ± 2.54	8.39 ± 4.69	1.7 ± 1.3	7.79 ± 5.92	2.63 ± 1.92	6.06 ± 4.41	6.66 ± 2.12	0.72 ± 0.73	0.004*
Urine protein	Nil		0 (0%)	0 (0%)	0 (0%)	0 (0%)	0 (0%)	1 (5%)	0 (0%)	2 (100%)	0.029*
	1+		0 (0%)	0 (0%)	1 (20%)	0 (0%)	3 (33.3%)	3 (15%)	0 (0%)	0 (0%)	
	2+		0 (0%)	1 (14.3%)	3 (60%)	1 (50%)	4 (44.4%)	3 (15%)	0 (0%)	0 (0%)	
	3+		1 (50%)	3 (42.9%)	1 (20%)	0 (0%)	1 (11.1%)	10 (50%)	4 (57.1%)	0 (0%)	
	4+		1 (50%)	3 (42.9%)	0 (0%)	1 (50%)	1 (11.1%)	3 (15%)	3 (42.9%)	0 (0%)	
Hematuria (n = 49)	Yes		0	3	5	1	7	19	1	2	<0.001*
	No		2	3	0	1	0	1	4	0	

The distribution of LN classes in the study cohort according to the ISN/RPS classification is presented in Table 4. A total of 20 cases of LN (37%) were identified in our study. Class IV LN was the predominant class (n = 10, 50%), followed by Class II LN (n = 6, 30%).

**Table 4:** Distribution of LN classes among the study cohort according to the ISN/RPS classification (n = 20) Data are presented as frequencies (n) and percentages (%). ISN/RPS: International Society of Nephrology/Renal Pathology Society, LN: lupus nephritis

LN ISN/RPS classification	Frequency (n)	Percent (%)
Class II	6	30
Class III	1	5
Class III + Class V	1	5
Class IV	10	50
Class IV + Class V	1	5
Class V	1	5

## Discussion

In this study, we assessed the clinical and laboratory parameters, indications for renal biopsy, and histopathological patterns observed in pediatric renal biopsies. The most common indication for renal biopsy was nephrotic syndrome, followed by nephritic syndrome. This finding is consistent with studies by Kanodia et al. [7] and Kumar et al. [10], who reported nephrotic syndrome as the most common indication for renal biopsy. In contrast, Dotis et al. [11] and Yen et al. [12] reported acute nephritic syndrome as the most common indication in their respective cohorts. In the present study, nephrotic syndrome included SRNS, steroid-dependent nephrotic syndrome with atypical features, and frequently relapsing nephrotic syndrome. Other indications included urinary abnormalities, AKI, hypertensive encephalopathy, systemic disease with renal involvement, and chronic kidney disease.

The mean age at renal biopsy in this study was approximately 10.7 years. In one of the largest Indian studies by Mohapatra et al. [13] involving biopsy-proven renal diseases, the mean age was 12.8 years. In a Croatian study by Arapovic et al. [9], the mean age of the cohort was 9.8 years. Regarding age and sex distribution, LN was more common among older children (>10 years), with a mean age of 14.8 years, whereas IgAN showed a male predominance. These findings were consistent with those of Yen et al. [12] from northern Taiwan, who reported a higher frequency of LN among older children and a male predominance among patients with IgAN.

Secondary GN was the most prevalent category in this cohort, accounting for 31 cases (57.41%). However, the finding that secondary GN predominated over primary GN should be interpreted with caution. In pediatric nephrology practice, typical steroid-sensitive nephrotic syndrome occurring between 1 and 10 years of age is generally managed empirically with corticosteroids without renal biopsy. Consequently, our study population represents a highly selected biopsy cohort enriched for atypical or treatment-resistant cases. It should not be interpreted as reflecting the true community-level epidemiology of pediatric kidney disease.

**Figure 2:**
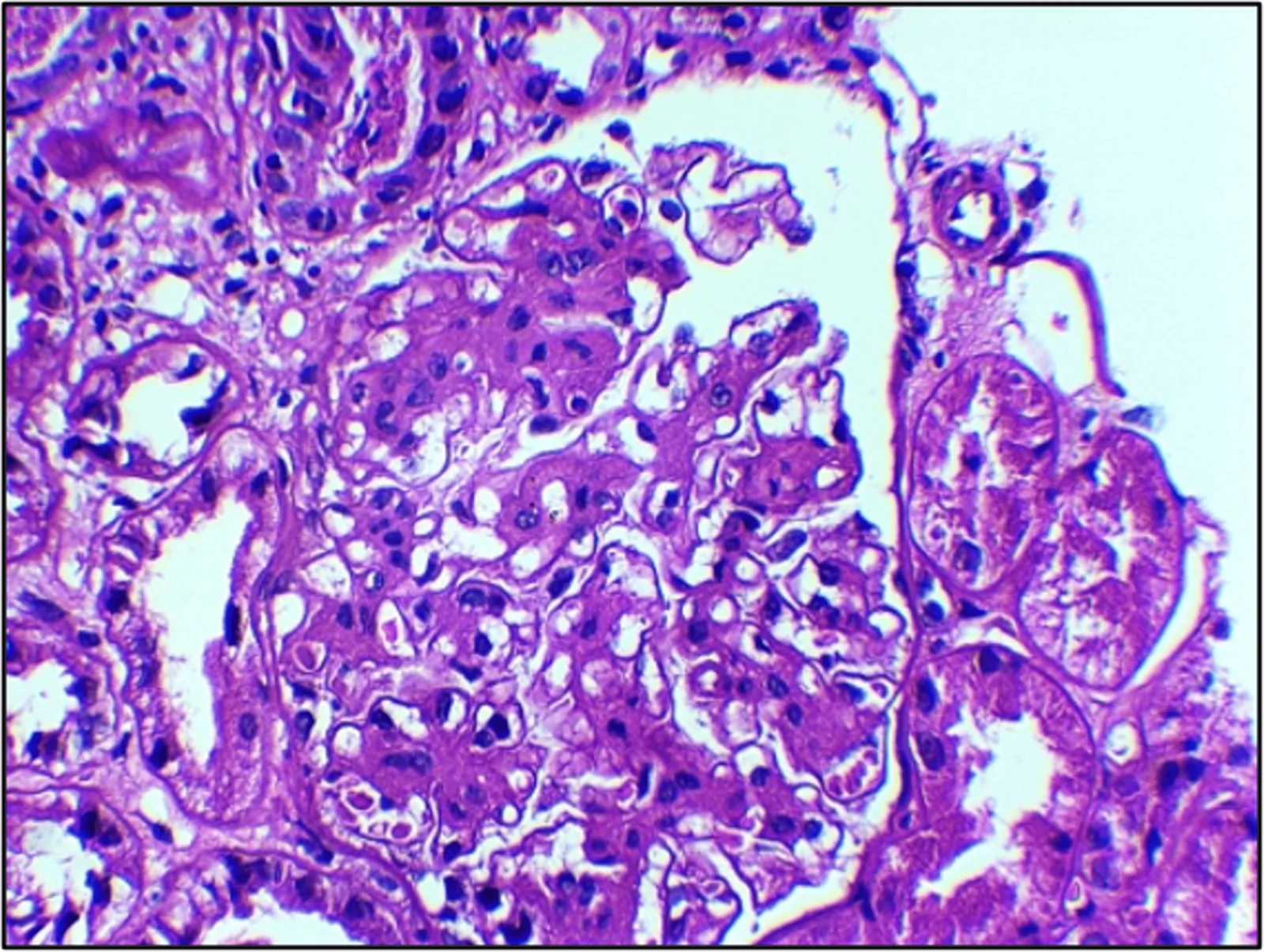
Class II LN showing mesangioproliferative GN (PAS stain, 40x) LN: lupus nephritis, GN: glomerulonephritis, PAS: periodic acid-Schiff Representative microscopic image from our study cohort.

**Figure 3:**
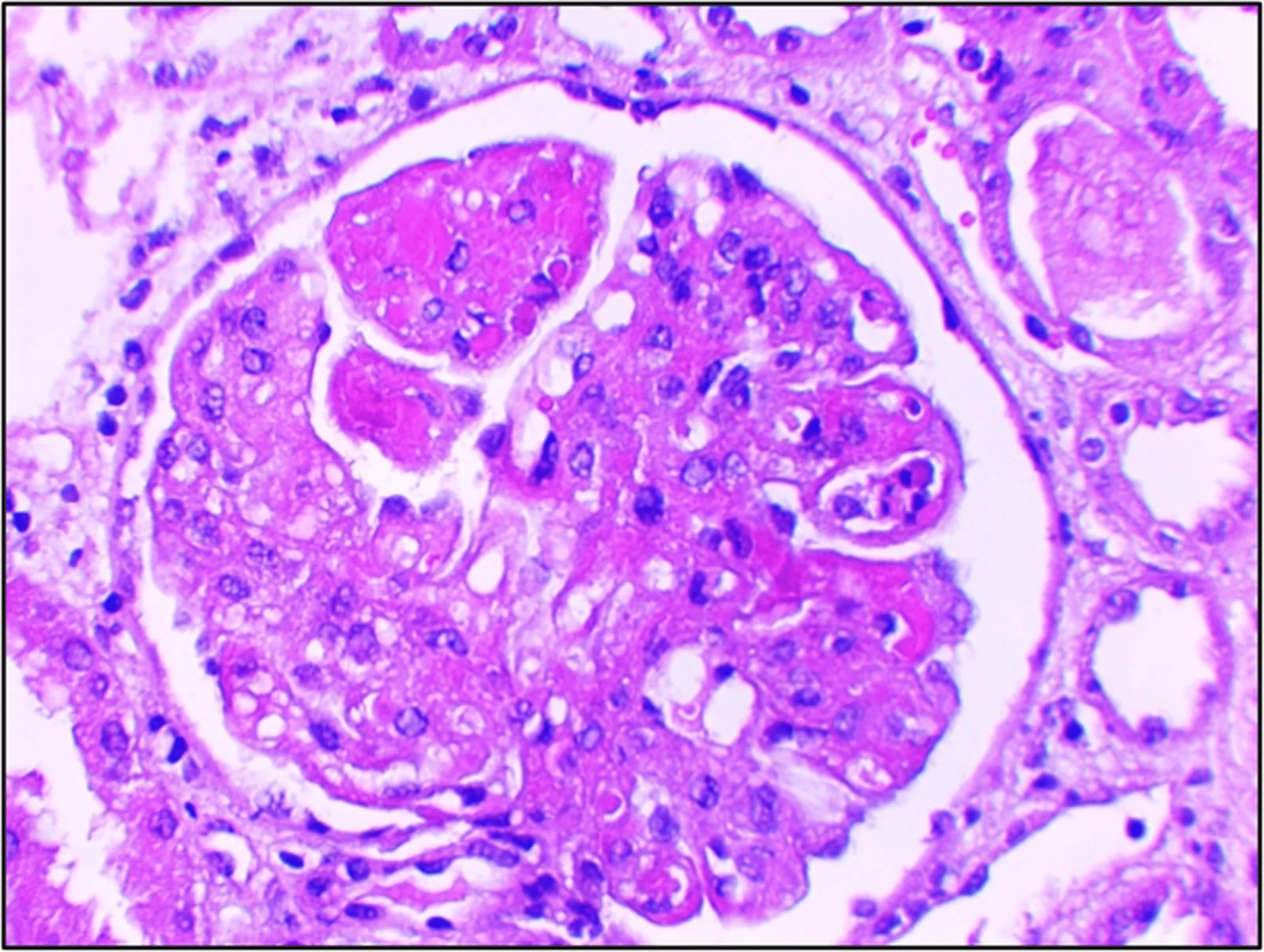
Class IV LN showing diffuse proliferative GN with hyaline thrombi (PAS stain, 40x) LN: lupus nephritis, GN: glomerulonephritis, PAS: periodic acid-Schiff Representative microscopic image from our study cohort.

**Figure 4:**
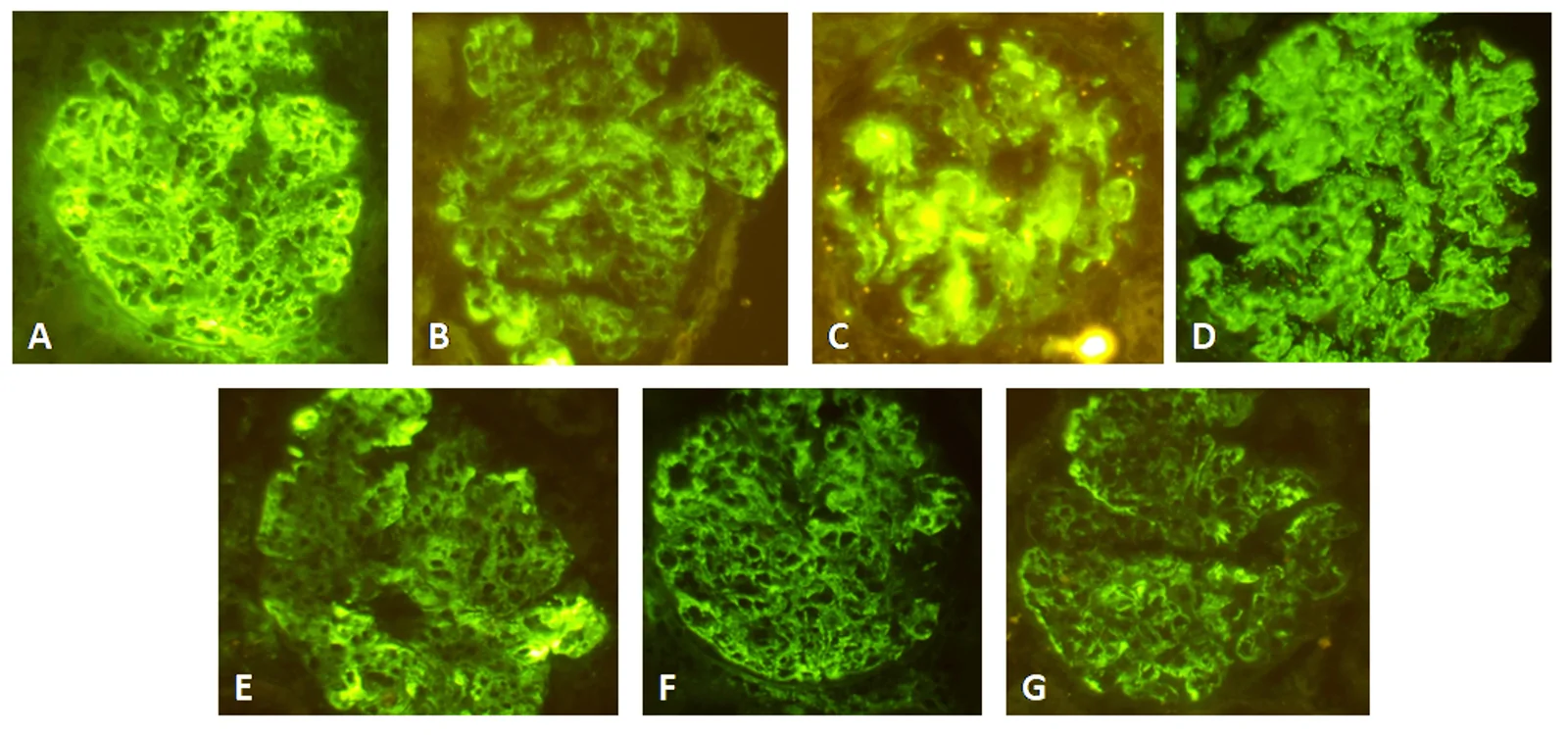
FITC staining in a case of LN A: IgG stain, B: IgA stain, C: IgM stain, D: C3 stain, E: C1q stain, F: kappa stain, G: lambda stain FITC: fluorescein isothiocyanate, LN: lupus nephritis, IgG: immunoglobulin G, IgA: immunoglobulin A, IgM: immunoglobulin M Representative microscopic images from our study cohort.

LN was the most common histopathological diagnosis in the cohort and the most frequent lesion among cases of secondary GN. Representative microscopic images of LN cases from our study are presented in Figures 2-4.

These findings are consistent with those reported in a recent Romanian study by Ciocanea et al. [14] and a Taiwanese study by Yen et al. [12], in which LN was reported as the most common form of secondary GN, accounting for 17.8% and 41% of cases, respectively. Childhood-onset SLE is more common among South Asian patients than in Western populations, with hematological, renal, and mucocutaneous manifestations being the most frequently affected [15]. In childhood-onset SLE, LN most commonly presents as diffuse proliferative GN, corresponding to Class IV LN. Over the past two to three decades, the proportion of pediatric patients diagnosed with LN has increased significantly, possibly attributable to changes in clinical practice and more frequent renal biopsies among patients with SLE, including those without overt renal manifestations [14].

In our cohort, diffuse proliferative GN, corresponding to Class IV LN, was the most common histological class, accounting for 50% of LN cases (n = 10). This finding is consistent with studies by Amatya et al. [16] and Lim et al. [17], who reported ISN/RPS Class IV LN as the most common class, with frequencies of 57.1% and 36.4%, respectively. Membranous Class V LN was identified in 5% of patients with LN (n = 1) as an isolated lesion and was also observed in combination with Class III and Class IV LN in one case each. In a study by Mutalik et al. [18], membranous LN was reported in 9.1% of the cohort. Pure membranous LN is uncommon in children and is often associated with proliferative GN. This may reflect more pronounced immune dysregulation, greater disease activity, and a higher propensity for inflammatory glomerular lesions in children.

**Figure 5:**
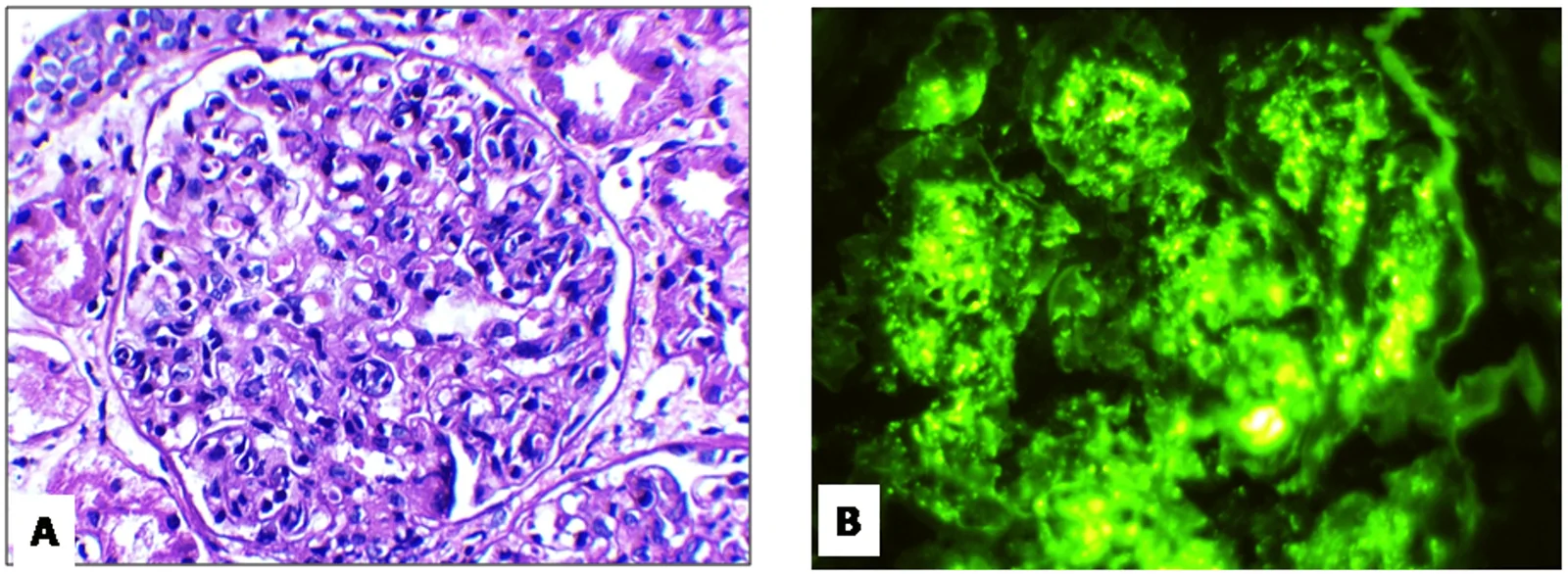
(A) Diffuse exudative GN in a case of IRGN (PAS stain, 40x) and (B) FITC staining showing a “starry-sky” pattern (C3 IF stain, 40x) GN: glomerulonephritis, IRGN: infection-related glomerulonephritis, PAS: periodic acid-Schiff, FITC: fluorescein isothiocyanate, IF: immunofluorescence Representative microscopic images from our study cohort.

IRGN (n = 9, 16.7%) was the most common primary GN in our cohort, followed by MCD (n = 7, 13%) and FSGS (n = 7, 13%), which occurred with equal frequency. This finding contrasts with those from most Indian cohorts, in which MCD has been reported as the most common diagnosis among primary GN cases. However, the frequency of IRGN in our cohort is comparable to that reported by Kanodia et al. [7] and Mohapatra et al. [13], who reported IRGN in 9.55% and 9.8% of cases, respectively. IRGN remains an important histopathological diagnosis in pediatric renal biopsy cohorts from developing countries. Representative microscopic images of IRGN cases from our study are presented in Figure 5.

IRGN is also frequently the most common histopathological lesion among children undergoing renal biopsy for acute nephritic syndrome. However, it is generally not the most frequent diagnosis in overall pediatric renal biopsy cohorts. In contrast, Western pediatric renal biopsy studies have reported IgAN, MCD, and FSGS as predominant diagnoses [19-20]. The higher proportion of IRGN in our cohort may reflect regional differences in disease epidemiology. Despite a global decline in IRGN in developed countries, it remains a significant burden in many low- and middle-income countries, where skin and upper respiratory tract infections remain prevalent. The predominance of IRGN in our cohort may therefore reflect both the persistent burden of infection-associated disease in the region and referral bias inherent to tertiary care centers.

**Figure 6:**
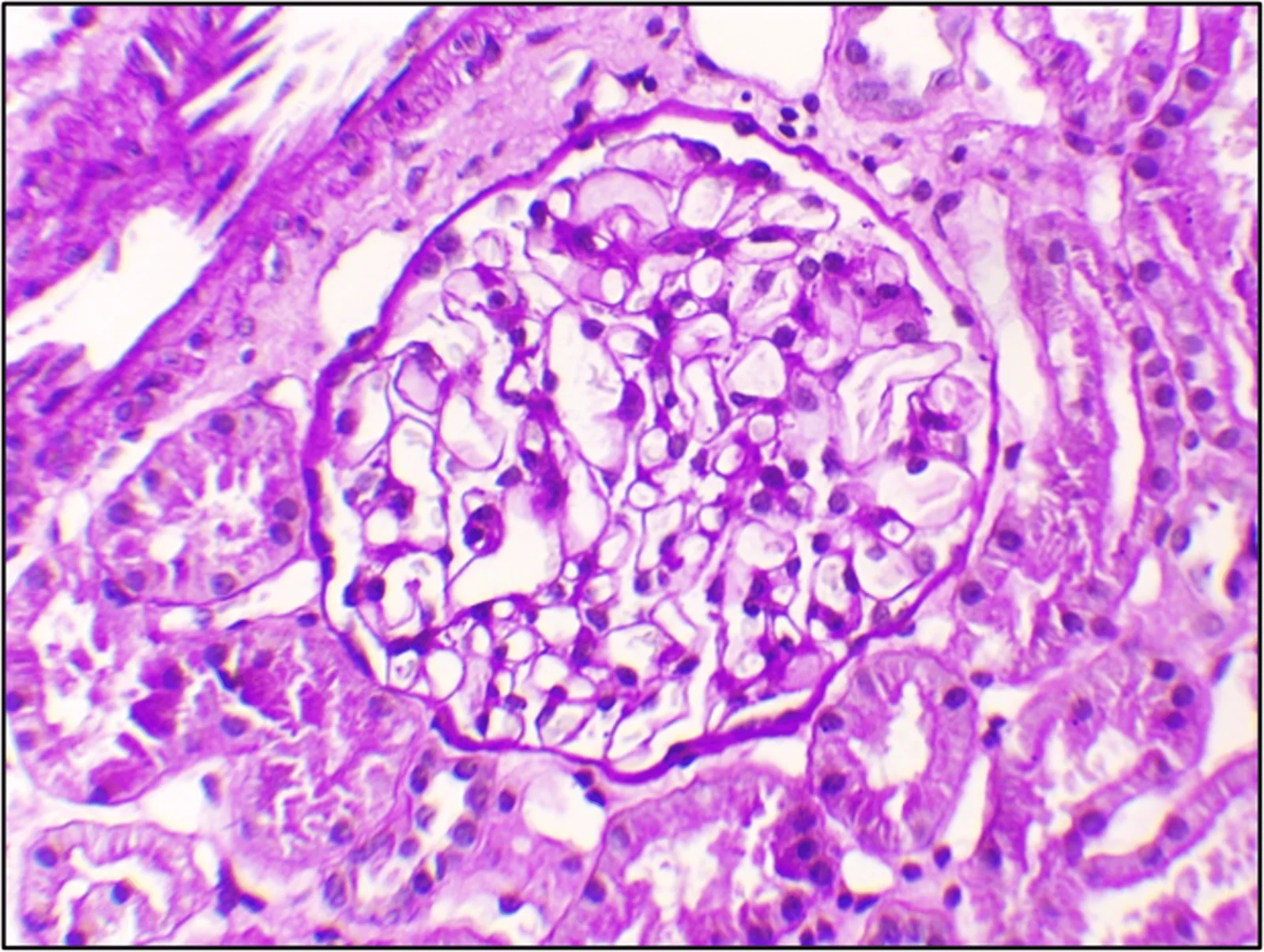
MCD showing glomeruli with preserved architecture and essentially normal appearance on light microscopy (PAS stain, 40x) MCD: minimal change disease, PAS: periodic acid-Schiff Representative microscopic image from our study cohort.

**Figure 7:**
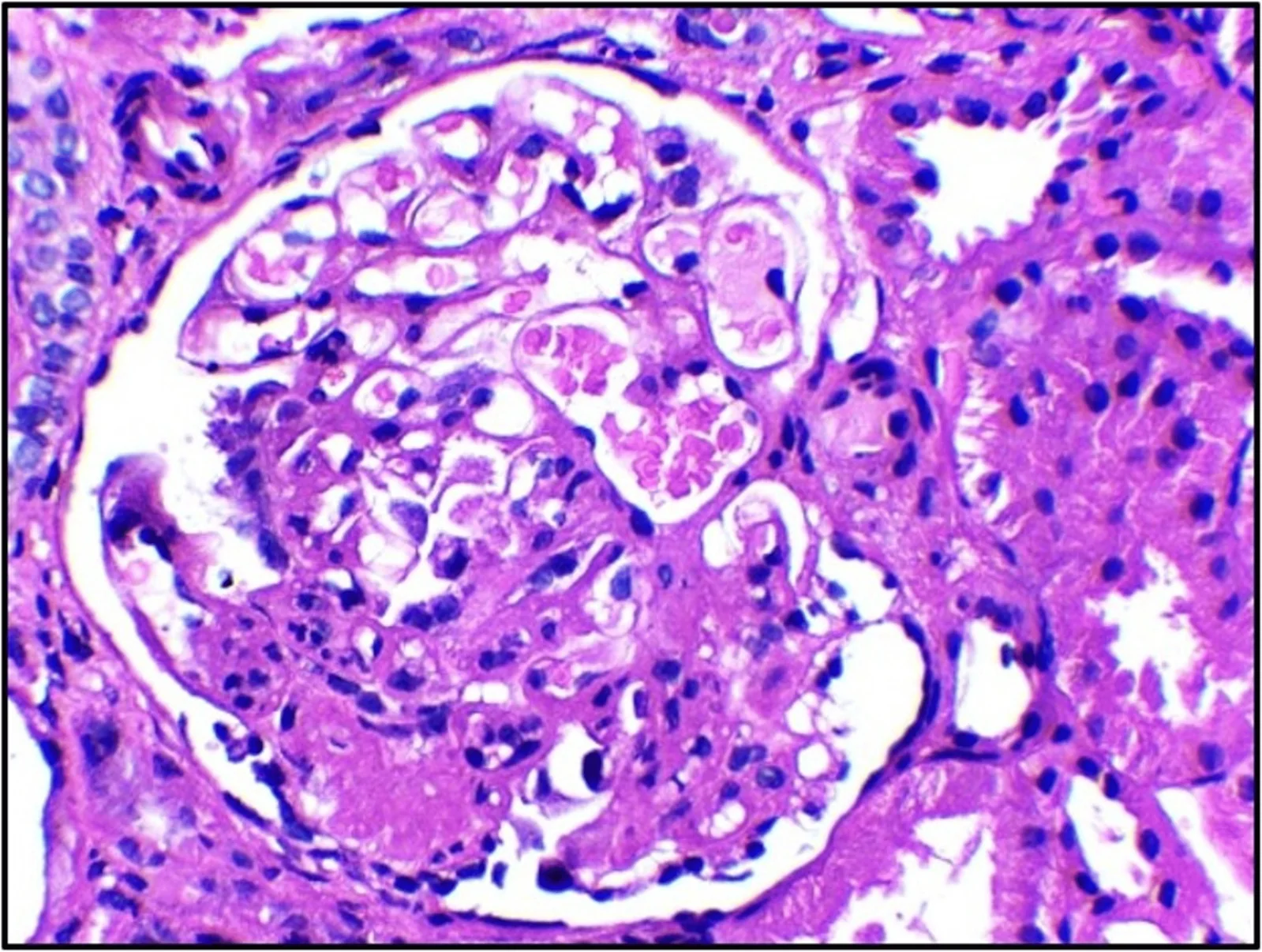
FSGS (PAS stain, 40x) FSGS: focal segmental glomerulosclerosis, PAS: periodic acid-Schiff Representative microscopic image from our study cohort.

MCD was the most common histopathological diagnosis among patients presenting with nephrotic syndrome, followed by FSGS. Representative microscopic images of MCD and FSGS cases from our study are shown in Figure 6 and Figure 7, respectively.

These findings are consistent with those of Yen et al. [12] and Mohapatra et al. [13], who also reported MCD as the most common histopathological diagnosis among patients presenting with nephrotic syndrome. Contemporary Indian pediatric renal biopsy studies suggest subtle but important changes in the histopathological spectrum over time. Although MCD has historically been the predominant lesion in childhood nephrotic syndrome, recent series have reported increasing frequencies of FSGS and other non-MCD lesions.

**Figure 8:**
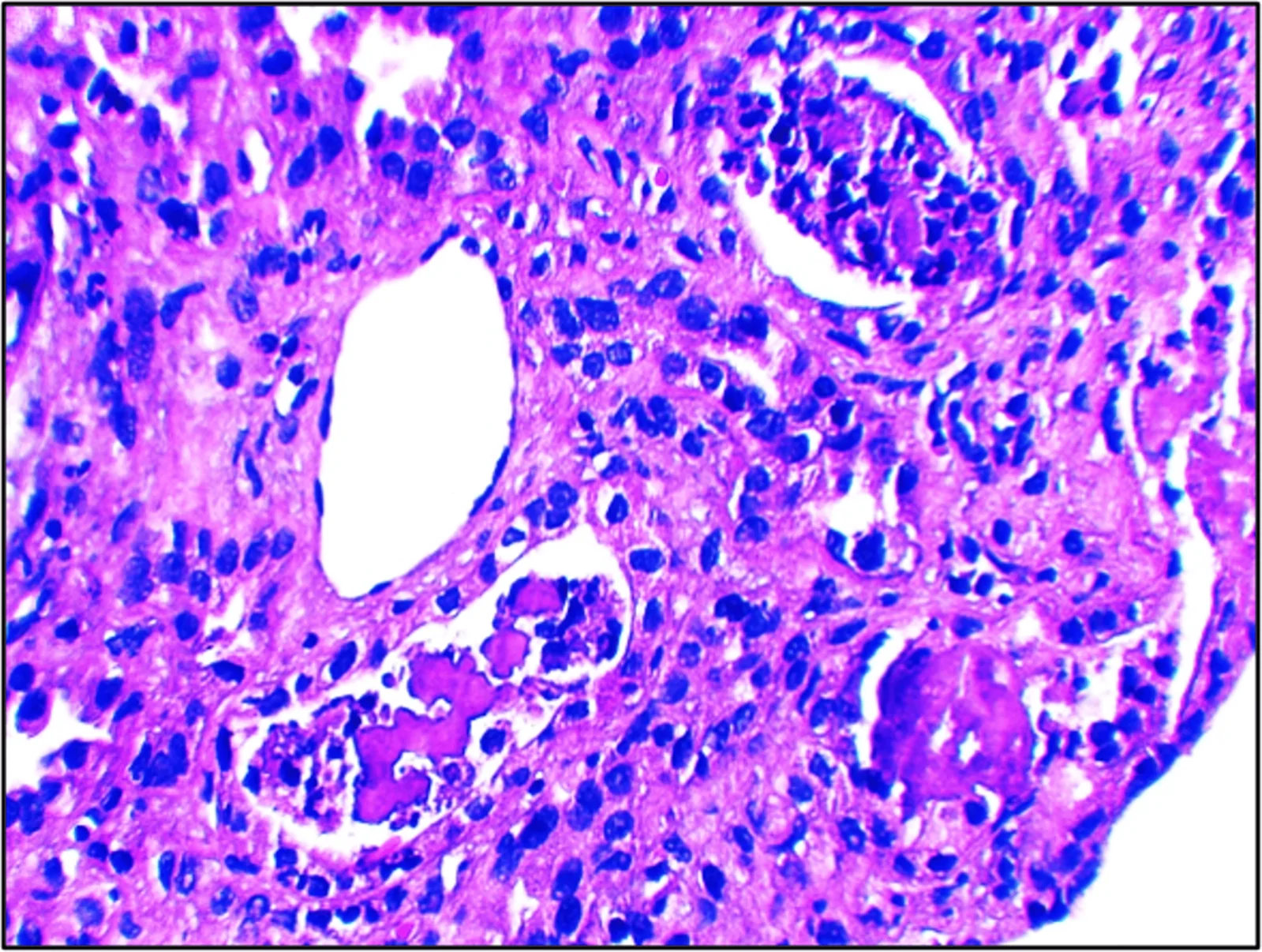
PCN showing globular pigmented casts within the tubular lumina (PAS stain, 40x) PAS: periodic acid-Schiff, PCN: pigment cast nephropathy Representative microscopic image from our study cohort.

**Figure 9:**
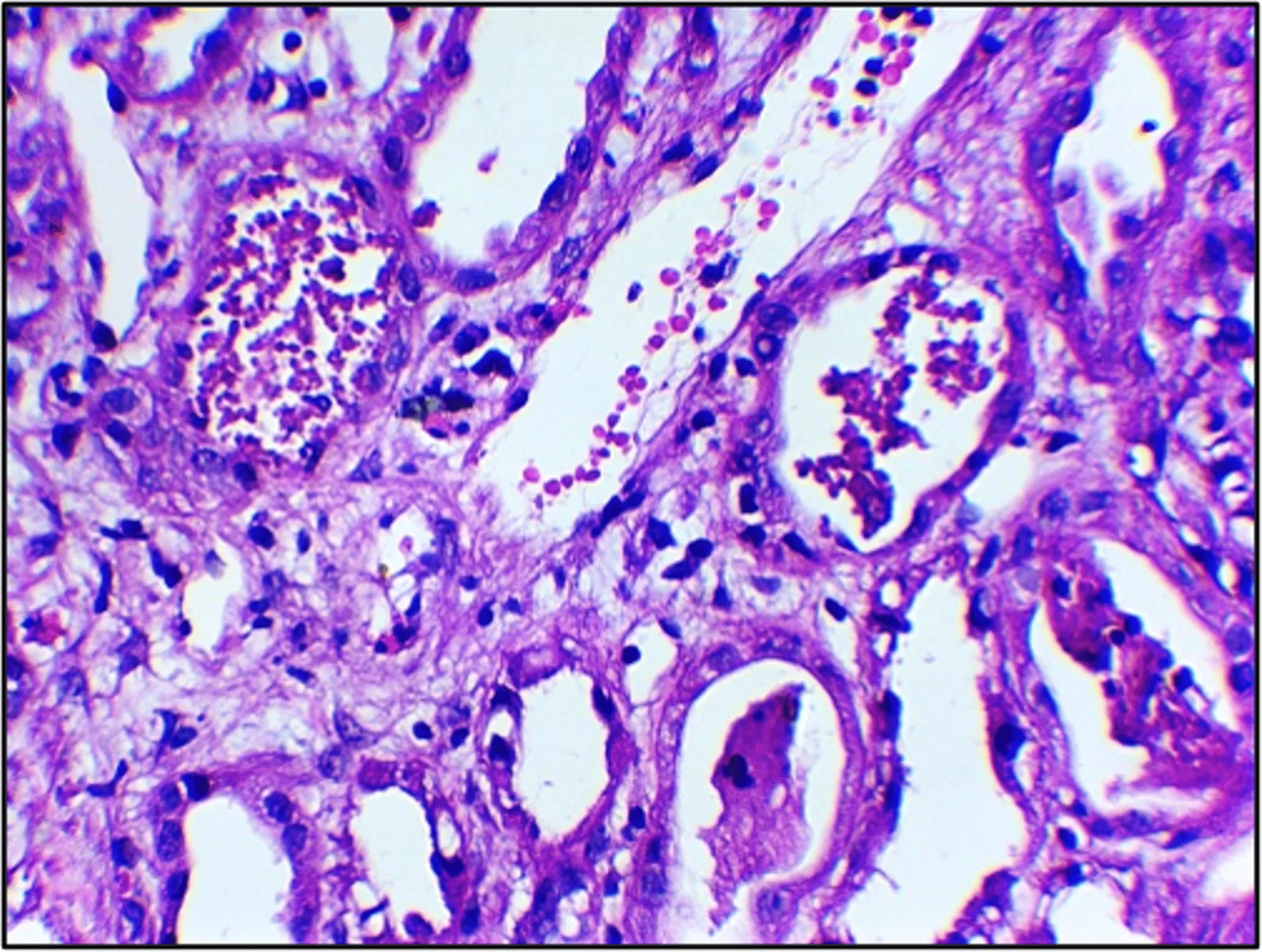
PCN showing granular casts in the tubular lumina accompanied by acute tubular epithelial injury (H&E stain, 40x) H&E: hematoxylin and eosin, PCN: pigment cast nephropathy Representative microscopic image from our study cohort.

Two cases of PCN (3.7%) were identified in our cohort. Representative microscopic images of PCN cases from our study are presented in Figures 8-9.

One case involved cerebral malaria with hemolysis, while the other patient presented with convulsions and was subsequently diagnosed with SLE with central nervous system involvement. PCN is rare in pediatric renal biopsy cohorts and is primarily described in isolated case reports and occasional cases within mixed-age biopsy series. The largest biopsy-based studies have originated from specialized renal pathology centers and have included only a small number of pediatric cases. PCN may result from myoglobin or hemoglobin casts, which can be differentiated using immunohistochemical (IHC) studies. IHC was not performed in these cases because of financial constraints. In a study by Patel et al. [21], rhabdomyolysis was the most common etiology of PCN.

Serum C3 levels differed significantly across histopathological diagnoses, with lower levels observed in patients with LN and IRGN than in those with MCD. This finding likely reflects differences in complement activation and consumption among disease entities, resulting in hypocomplementemia. Hypocomplementemia is a well-recognized feature of active LN and results from immune complex-mediated activation of the classical complement pathway, leading to complement consumption. Low serum C3 levels have also been associated with greater disease activity and severity [22]. Serum C4 levels also differed significantly across diagnostic categories, particularly between LN, MCD, and FSGS. Reduced C4 levels in LN likely reflect activation of the classical complement pathway and subsequent complement consumption [23]. In contrast, MCD, a primary podocytopathy characterized by minimal immune complex deposition and complement activation, typically exhibits normal serum C3 and C4 levels [24]. These findings suggest that serum C3 and C4 levels should be interpreted as supportive laboratory findings in conjunction with the clinical presentation and histopathological features rather than as independent diagnostic markers.

Limitations of the study

A major limitation of this study is the lack of electron microscopy, which is an important adjunct in the evaluation of glomerular diseases. Although diagnoses were established using light microscopy and immunofluorescence, the absence of electron microscopy may have affected diagnostic accuracy in selected cases, particularly those requiring ultrastructural assessment for definitive classification, such as differentiating MCD from early FSGS. The study is also subject to case selection and referral bias because it was conducted at a tertiary care center, where patients with more complex or severe disease are more likely to be referred and undergo renal biopsy. Consequently, the findings may not be fully generalizable to the broader pediatric population.

## Conclusions

This study demonstrates that nephrotic and nephritic syndromes were the most common indications for pediatric renal biopsy. Secondary GN was more prevalent than primary GN. LN was the most frequent histopathological diagnosis, with Class IV LN accounting for half of the cases. Among primary glomerular diseases, IRGN was the most common diagnosis, followed by MCD and FSGS.

Although the predominance of nephrotic syndrome as an indication for renal biopsy is consistent with findings from other Indian studies, the high frequency of LN and IRGN may reflect regional epidemiological patterns that differ from those reported in Western pediatric biopsy cohorts. These findings underscore the importance of renal biopsy for establishing accurate diagnoses and guiding the management of pediatric renal diseases. Significant differences in serum C3 and C4 levels were observed across histopathological diagnoses. Lower C3 levels in LN and IRGN and lower C4 levels in LN support the role of complement consumption in immune complex-mediated renal injury. Serum complement levels should therefore be interpreted as supportive laboratory findings in conjunction with clinical and histopathological findings rather than as independent diagnostic markers.
